# Supplementary material for: Sparsified federated learning with differential privacy for intrusion detection in VANETs based on Fisher Information Matrix
Source: PLoS One. 2024 Apr 17;19(4):e0301897. doi: 10.1371/journal.pone.0301897 (PMC11023508; doi:10.1371/journal.pone.0301897)
Supplement: Attachment — Submitted filename: Response to Reviewers.docx [file pone.0301897.s001.docx]

Dear Editor,

We are immensely grateful for the opportunity to revise our manuscript. It is with great pleasure that we submit the revised version entitled "Sparsified Federated Learning with Differential Privacy for Intrusion Detection in VANETs based on Fisher Information Matrix" for your consideration.

During the revision process, we have conducted all the suggested experiments, performed thorough analyses of related work, and made substantial clarifications where the content was previously unclear. We have addressed all the comments raised by the reviewers and yourself, which are elaborated on in the "Rebuttal Letter" attached to this correspondence.

Aware of the high standards PLOS ONE upholds for article quality, we have also proactively refined areas not directly pointed out by the reviewers. These include enhancing the clarity of the introduction, and unifying the methodological explanations. The extent of these revisions can be seen in the "Revised Manuscript with Track Changes", also attached.

All authors have read and consented to the resubmission of this manuscript. Should you have any questions or require further information, please feel free to contact me.

Thank you for your consideration of our paper and we are looking forward to hearing from you!

Yours sincerely,

Rui Chen

Dalian University of Technology

Email:chenrui_dut@163.com

**Rebuttal Letter**

**Reviewer 1:**

This paper introduces FFIDS, a novel approach for enhancing the security of VANETs through the integration of Federated Learning (FL) and Differential Privacy (DP). The primary focus is on addressing the challenge of preserving privacy in FL while minimizing the impact on model accuracy and communication costs. FFIDS employs a unique combination of model parameter pruning based on the Fisher Information Matrix and DP techniques. By identifying and pruning unimportant parameters, it reduces privacy budget consumption per iteration without sacrificing accuracy. DP noise is selectively added to submodels, prioritizing important parameters, thereby optimizing privacy preservation. The proposed approach is validated through extensive experiments on public and simulation datasets, demonstrating its effectiveness and superior performance in VANET intrusion detection models. However, some issues may need more attention.

**Comment 1:** Line 21 of the manuscript should be revised to replace "Inference attacks" with "member inference attacks." Additionally, there is a grammar mistake on line 14 that needs correction.

**Response 1:** Thank you for the valuable suggestions from the reviewers. Following your guidance, we have precisely corrected "Inference attacks" to "member inference attacks" to enhance the accuracy of terminology. In addition, regarding the grammatical issue in the second paragraph at Line 14, we have made the corresponding corrections. Moreover, we have specifically invited a native English speaker who is a professional editor to thoroughly polish the language throughout the paper.

**Comment 2:** Line 189, should "g(w, D)" be changed to "s(w, D)"?

**Response 2:** Thank you very much for the meticulous modification suggestions from the reviewers. We did find some mathematical formulas that were not accurate enough during our review process. We have now comprehensively revised these formulas in the paper. In the updated version, "g(w, D)" has been corrected to "s(w, D)" to ensure consistency and accuracy of the notations.

**Comment 3:** Line 211, should "Z[j]" be changed to "Z[i]"?

**Response 3:** We are grateful for the reviewer's comments regarding revisions, and we have changed "Z[j]" to "Z[i]" accordingly.

**Comment 4:** Line 218, should "wi" be changed to "w[i]"?

**Response 4:** We greatly appreciate the valuable feedback provided by the reviewer. Upon careful examination, we recognized that the parameter notation "wi" might cause confusion for readers. To address this, we have corrected all instances of "wi" to "w[i]," thereby improving the clarity and consistency of the formulas. Similarly, we noted analogous issues in Equation 3 and Algorithm 2, and have made the corresponding adjustments.

**Comment 5:** Is the loss function "P(y|x; w)" or "f(w)"?

**Response 5:** We extend our sincere thanks to the reviewer for their meticulous review and invaluable suggestions. We have noticed that the original notation "P(y|x; w)" used to represent the loss function could potentially lead to misunderstandings among readers. Therefore, we have revised the loss function references in Sections 3.2 and 4.1, as well as in Algorithms 1 and 2, and Section 4.3. The loss function is now denoted by "Loss(f(y|x; w))", where "f(y|x; w)" represents the model function. We are genuinely appreciative of the reviewer's careful examination, which has significantly enhanced our paper.

**Comment 6 (1):** In the experimental section, there are a few issues may need further considerations: (1) Firstly, Table 2 should not exceed the length specified for the main text. Secondly, the data in Table 2 appears perplexing. Why are only NbAFL, DPFL-SR, and FFIDS included as methods? Additionally, there is no presentation of comparative results for communication overhead based on matrix update frequencies. Finally, I am particularly interested in how communication overhead is measured.

**Response 6 (1):**

Regarding issue 1, we are deeply grateful for the valuable advice provided by the reviewer. Upon re-examination, we indeed found oversights. We have now reorganized the data in the tables and made corresponding format modifications to ensure the accuracy of the data and the clarity of the tables.

**Revised Table:** Table 2.

In response to issue 2, we are very thankful for the valuable comments raised by the reviewer. In the federated learning training process studied, the proportion of model pruning employed in each training epoch was indeed consistent. The communication overhead considered in this paper refers to the communication costs incurred by a single client during a federated learning training cycle; therefore, the overhead of update frequency was not included. Based on your suggestion, we will clarify this point further in the paper to prevent any misunderstanding by the readers regarding the method for computing communication overhead.

**Revised Content:** Section 6.3, first paragraph.

As for issue 3, we express our sincere appreciation for the reviewer's thorough review and valuable feedback. In the federated learning training scenario, we measure the communication overhead by directly quantifying the storage space occupied by the client-uploaded models received by the central server. To eliminate any potential queries from the readers, we have supplemented the experimental section with the specific measurement method for communication overhead. We thank the reviewer once more for their valuable input on our work.

**Revised Content:** Section 6.3, first paragraph.

**Comment 6 (2):** In Figure 4, why does LDP-FedSGD outperform FFIDS? LDP-FedSGD does not involve parameter selection, and my understanding is that this approach should introduce more negative impacts. I am also puzzled by the fact that LDP-FedSGD in Figure 5 seems unaffected by the privacy budget.

**Response 6 (2):**

We thank the reviewer for their valuable comments. In response to issue one, we have revised the relevant experimental sections to eliminate issues that could potentially confuse readers. We have elaborated in detail within the article the performance advantages of LDP-FedSGD over FFIDS, and we clearly state that although model pruning can reduce communication costs during distributed training, the proportional reduction of the model in the pruning process will inevitably lead to the loss of model weights. In contrast, LDP-FedSGD does not employ gradient pruning throughout the training process, thereby avoiding accuracy degradation due to loss of gradients. As demonstrated in the revised Figure 5 (originally Figure 4), the efficacy of LDP-FedSGD surpasses that of FFIDS, which is consistent with theoretical expectations. The model inevitably sacrifices some accuracy to reduce communication overhead, resulting in decreased final model performance.

**Revised Content:** Section 6.5, paragraph e).

Regarding issue two, we acknowledge that the F1 scores of LDP-FedSGD displayed in Figure 6 (originally Figure 5) appear unaffected by changes in the privacy budget, which could lead to confusion among readers. In reality, the implementation of the LDP-FedSGD algorithm in our experiments did not incorporate differential privacy noise, which explains why its F1 score is insensitive to the privacy budget. We are grateful for the reviewer's valuable suggestion and have made detailed revisions to the description of the experiments to ensure that readers do not misunderstand this aspect.

**Revised Content:** Section 6.6, first paragraph

**Comment 6 (3):** The confusion matrix should be added in order to verify the results of performance metrics.

**Response 6 (3):**

We are sincerely grateful for the valuable comments provided by the reviewer. This research work focuses on a strategy for pruning model gradients using information matrices in federated learning. In our initial study, we compared four baseline models, among which the DPFL-SR method shares some similarities with the pruning principle proposed in this paper. Following the reviewer's guidance and our subsequent review, we recognized the need to compare our method with other strategies for model lightweighting.

Accordingly, we have added a comparison of two quantization-based model gradient lightweighting methods, cpSGD and QuAsncFL, and included DPFL-SR as a benchmark. To more closely reflect the performance of real vehicular-level embedded devices, we selected the Raspberry Pi 4B 4G as our vehicular-level test platform. On this platform, we conducted federated learning training experiments for the intrusion detection model using the chosen gradient lightweighting algorithms. This approach will help in the assessment and demonstration of the effectiveness of each algorithm in actual vehicular environments.

**Added Content:** Section 6.4

**Reviewer 2:**

This paper presents a privacy-preserving and communication-efficient Federated Learning algorithm for intrusion detection in VANETs. The primary concept revolves around utilizing the Fisher Information Matrix to evaluates the importance of model parameters, further pruning the model to retain important model parameters and adding DP noise only into these important parameters for diminishing the private budget consumption. Preliminary experiments are conducted to demonstrate the efficacy of the proposed approach on two public datasets and two F2MD simulated datasets. The suggested approach is acceptable, albeit with several critical concerns that require attention:

**Comment 1**: due to the involvement of gradient pruning in the proposed method, it is necessary to include relevant content in order to align the gradient pruning method with theoretical foundations in Section 3

**Response 1:**

We extend our heartfelt thanks to the reviewer for their constructive feedback. Regarding the gradient pruning algorithm, we recognize in Section 3.2 that the original narrative could potentially lead to ambiguity for the readers. In response, we have thoroughly revised this section and supplemented it with the motivation and theoretical basis for the gradient pruning algorithm. Furthermore, we have changed the title of Section 3.2 to "Gradient Pruning for Fisher Information Matrix" to facilitate a smoother reading experience for our audience.

**Revised Content:** The first and second paragraphs of Section 3.2, as well as the title of Section 3.2.

**Comment 2**: Please note that there is a error in Table 2 of the experimental results.

**Response 2:**

We express our gratitude to the reviewer for their valuable suggestions. Upon re-evaluation, we have identified oversights in our work. We have now reorganized the data in the tables and made corresponding adjustments to the format of the tables to ensure the accuracy of the data and the clarity of the presentation.

**Revised Table:** Table 2

**Comment 3:** It is advisable to provide meaningful citation locations for references, for example, a method [21]. Please check for duplicate citations in the order of references.

**Response 3:**

We greatly appreciate the reviewer's comments. Regarding the issue of duplicate citations in the text, we have conducted a comprehensive revision.

**Comment 4:** Finally, please improve the quality of the paper by correcting grammatical errors and spelling mistakes, such as a spelling error in the left image of Figure 1.

**Response 4:**

We are deeply grateful for the reviewer's valuable feedback. We have engaged a native English-speaking teacher to thoroughly proofread and polish the manuscript. We extend our heartfelt thanks to the reviewer for their meticulous review, which has significantly helped us improve the paper.
